# ApoE4 lowers age at onset in patients with frontotemporal dementia and tauopathy independent of amyloid-β copathology

**DOI:** 10.1016/j.dadm.2019.01.010

**Published:** 2019-03-19

**Authors:** Carolin Koriath, Tammaryn Lashley, William Taylor, Ronald Druyeh, Athanasios Dimitriadis, Nicola Denning, Julie Williams, Jason D. Warren, Nick C. Fox, Jonathan M. Schott, James B. Rowe, John Collinge, Jonathan D. Rohrer, Simon Mead

**Affiliations:** a UCL Institute of Prion Diseases, London, UK; b Queen Square Brain Bank for Neurological Disorders, Department of Movement Disorders, UCL Institute of Neurology, London, UK; c UK Dementia Research Institute at Cardiff University, Cardiff, UK; d Dementia Research Centre, Department of Neurodegenerative Disease, UCL Institute of Neurology, Queen Square, London, UK; e UK Dementia Research Institute at UCL, London, UK; f Department of Clinical Neurosciences, University of Cambridge, UK; g Medical Research Council Cognition and Brain Sciences Unit, Cambridge, UK

## Abstract

**Introduction:**

Apolipoprotein E (ApoE) is the most important genetic risk factor for Alzheimer's disease (AD), with ApoE4 thought to enhance and accelerate amyloid-β (Aβ) pathology. ApoE4 has recently been described to increase neurodegeneration in a mouse model of frontotemporal dementia (FTD), *in vitro*, and in patients, demonstrating that ApoE4 modifies tauopathy independently of Aβ. This raises the question whether ApoE genotype also modifies the clinical phenotype in patients with FTD with tau pathology.

**Methods:**

We analyzed 704 patients with FTD, including a genetically and neuropathologically confirmed subset, and 452 healthy elderly controls. We compared ApoE4 genotype frequency and age at onset in tau+ or TDP43+ FTD patients with or without Aβ copathology.

**Results:**

The ApoE4 genotype lowered age at onset in patients with FTD and tau pathology, particularly once accounting for confounding effects of Aβ pathology.

**Discussion:**

We conclude that ApoE4 accelerates neurodegeneration in FTD patients with *MAPT* mutations or FTLD-tau pathology, independent of Aβ.

Apolipoprotein E (ApoE) is the strongest known common genetic risk factor for Alzheimer's disease (AD) [1], [2]. Relative to the most common allele (ApoE3), ApoE4 is associated with an increased risk of late-onset AD and an earlier age at onset (AAO); conversely, ApoE2 confers lower risk and later onset [2], [3]. Multiple lines of evidence suggest that ApoE4 risk in AD principally relates to enhanced and accelerated cerebral Aβ pathology [4]. More precisely, ApoE4 appears to accelerate the early seeding of amyloid pathology, most likely by decreasing Aβ clearance and enhancing Aβ aggregation [5]. Shi et al*.* recently described how ApoE4 increases the burden of cerebral tau pathology, neuroinflammation, and brain atrophy in a P301S mouse model of frontotemporal dementia (FTD) and *in vitro*; they also demonstrated that in patients with a primary tauopathy, ApoE4 was associated with more severe regional neurodegeneration and that ApoE4+ AD patients with amyloid-β (Aβ) pathology showed faster disease progression [6]. New evidence from Shi et al*.* demonstrates that ApoE4 modifies tauopathy independent of Aβ, raising the question whether ApoE genotype also influences risk or modifies the clinical phenotype in patients with primary tauopathies. Several studies report an association between ApoE and FTD with a protective effect for ApoE2 and an increased risk conferred by ApoE4 [7], [8], [9], whereas another shows no association [10]. A recent publication demonstrated a deleterious effect of ApoE2 in a tau transgenic mouse model [11]. However, interpretations of the associations of ApoE with the clinical syndrome of FTD are complicated by two factors: frontal variant AD can be misdiagnosed for FTD, and the fact that the FTD syndrome comprises tau, TDP-43, or other pathologies. Prompted by Shi et al. [6], we therefore analyzed existing data we held on 704 patients with FTD and 452 healthy elderly controls using SPSS25 to test the hypothesis that ApoE genotype has a modifying effect on clinical phenotype in those with or expected to have tau pathology defined by a highly penetrant *MAPT* gene mutation or by neuropathological examination. We found that the ApoE4 genotype lowered age at clinical onset in patients with dementia and tau pathology, and was a particularly strong effect once the confounding effects of amyloid β pathology were taken into account.

The patients in this FTD cohort were previously tested for causative genetic mutations [12] and are described in Table 1. Patients with causative mutations in genes not typically associated with FTD were excluded from the present analysis. Patients were analyzed in groups based on genetic and/or neuropathological data, patients in whom no causative mutation had been identified and for whom no neuropathological data were available either were classified as “clinically diagnosed” and not included in analyses of genetic subgroups. Dementia and personality change were the predominant symptoms in our series (54.7% - 33.3% “behavioral type FTD”, 21.4% “FTD”, 1.1% “Dementia”), followed by aphasia (31.8% - 16.9% progressive nonfluent aphasia, 13.1% semantic dementia, 1.8% logopenic progressive aphasia) and additional motor or muscular symptoms (11.5% - 6.0% corticobasal degeneration, 2.4% progressive supranuclear palsy, 3.7% FTD with motor neuron disease, 0.3% inclusion body myositis with Paget's disease and FTD). As has been shown previously [7], ApoE4 was more common in clinically diagnosed FTD compared with healthy elderly controls; 221 patients (31.4%) and 105 controls (23.2%) were at least heterozygous for ApoE4 (chi-squared test, *P* = .003), carrying either one or two ApoE4 alleles. Based on Shi et al*.* [6], we hypothesized that ApoE4 would have a modifying effect on AAO in FTD cases with proven or known or expected tau pathology. While AAO is known to be highly correlated among family members with a highly penetrant *MAPT* gene mutation [13], here, AAO in confirmed FTD patients who had *MAPT* gene mutations or FTLD-tau neuropathology was significantly lower in the presence of an ApoE4 allele (tau+ ApoE4+ compared with tau+ ApoE4− patients; 53.0 years vs. 56.9 years, t-test, *P* = .043, Fig. 1A). In a further exploratory analysis, we tested whether ApoE4 also affected non-tau cases in the same way. However, ApoE4 neither had modifying effect on AAO in FTD patients with FTLD-TDP43 neuropathology or in FTD patients without an ascertained cause, nor did sex affect AAO in the different groups (TDP+ ApoE4+ compared with TDP+ ApoE4−: 57.6 years vs. 58.1 years; clinical FTD ApoE4+ compared with clinical FTD ApoE4−: 58.7 years vs. 58.9 years; ANOVA, all *P* values > 0.05; all Fig. 1A).

**Table 1:** Characteristics of patients with frontotemporal dementia (FTD) and healthy elderly controls

	All FTD	Clinical FTD	Confirmed FTD	FTD - tau	FTD - TDP	Controls
N	704	449	255	104	140	456
AAO (years)	58.1	58.9	56.4	55.7	58.1	76.6
N Mutation	-	-	166	53	106	-
N Neuropathology	-	-	139	70	65	-
N ApoE4+	220	139	81	32	44	105
ApoE4+ AAO (years)	57.5	59.1	54.9	53.0	58.0	75.2
ApoE4- AAO (years)	58.3	58.9	57.4	56.9	58.1	77.1
N Aβ+	-	-	44	26	18	-
AAO Aβ+	-	-	61.0	60.2	62.2	-
AAO Aβ-	-	-	57.1	56.9	58.4	-
AAO (N) ApoE4+/Aβ -	-	-	51.4 (12)	49.3 (6)	56.2 (5)	-
AAO (N) ApoE4-/Aβ -	-	-	58.1 (71)	58.4 (31)	58.7 (37)	-
AAO (N) ApoE4+/Aβ+	-	-	56.1 (23)	54.9 (15)	58.0 (8)	-
AAO (N) ApoE4-/Aβ+	-	-	66.3 (21)	67.0 (11)	65.6 (10)	-

NOTE. AAO refers to age at clinical onset, given in years of age; N refers to number of cases. “All FTD” comprises all 704 patients with FTD included in this study; these were all patients from the UCL FTD cohort except patients previously shown to carry mutations not typically associated with FTD [12]. “Clinical FTD” refers to the 449 patients with a clinical diagnosis of FTD but without a genetic diagnosis despite previous testing on a dementia panel [12] and no available neuropathological data.

NOTE. “Confirmed FTD” comprises 255 patients with a genetic or neuropathological confirmation of their diagnosis or both. It includes 59 patients with *C9orf72* expansions, 53 patients with *MAPT* mutations, 44 patients with *GRN* mutations, five cases with *VCP* mutations, two cases with *TBK1* mutations, as well as one case each with a mutation in *CHMP2B*, *TYROBP*, and *TARDBP* mutation. “Confirmed FTD” also includes 139 patients with neuropathologically confirmed disease: 70 patients with tau pathology and 65 patients with TDP-43 pathology, as well as 4 neuropathologically confirmed FUS cases. 19 patients with neuropathologically confirmed tau pathology also had a genetically confirmed deleterious MAPT mutation, whereas 31 patients with neuropathologically confirmed TDP-43 pathology were found to also carry a deleterious mutation (13 *C9orf72* expansion, 17 *GRN* mutations, 1 *TBK1* mutation). Patients with both genetically and neuropathologically confirmed disease were only counted once, explaining disparities of sums in the table. “N Mutation–Tau” refers cases due to causal mutations in the *MAPT* gene. “N Mutation–TDP” refers to causal mutations in the *C9orf72* (59 cases), *GRN* (44 cases), *TARDBP* (one case) and *TBK1* (2 cases) genes, which are known to cause TBK-43 pathology. “N Mutation–Confirmed FTD” comprises all genetically confirmed cases as described previously. Subcohorts referring to Aβ only include cases with available neuropathological data. For 7 patients with tau pathology and 5 patients with TDP pathology, information on Aβ status was not available; for one tau patient, information on AAO was not available. AAO is given in years, and in controls refers to age at testing. ApoE4+ includes all carriers of at least one ApoE4 allele, both homo- and heterozygous.

**Fig. 1:**
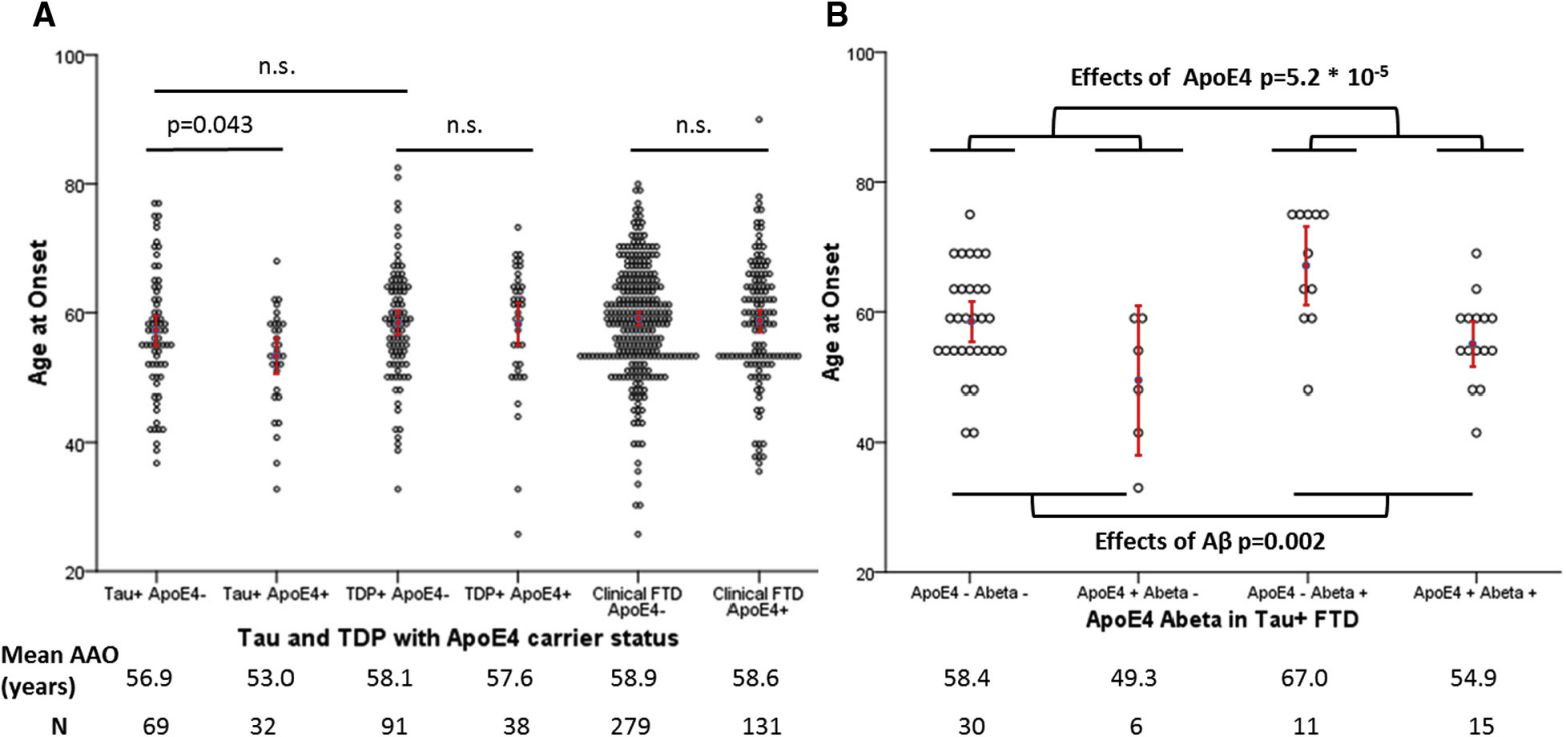
ApoE4+ status is associated with significantly lower age at onset (AAO) in FTD cases with *MAPT* mutations or FTLD-tau neuropathology, but not in those with TDP pathology or in those without an ascertained genetic or neuropathological diagnosis (clinical FTD) (A). ApoE4 carrier status is associated with lower AAO, whereas Aβ copathology was associated with later AAO (B). (B) displays the *P* values for the regression coefficients ApoE4 and Aβ. Each dot corresponds to the AAO of one case, with the mean and 95% confidence interval marked in red. The number of patients in each subcohort is given in line *N*. For (A), AAO was not available for 3 tau+ ApoE4− patients, 4 TDP+ ApoE4− and 6 TDP+ ApoE4+ cases, as well as 31 clinical FD ApoE4− and 9 clinical FTD ApoE4+ patients; for Figure 1B, 62 FTD tau+ patients with available ApoE4 status and neuropathological data were included; AAO was not available for 1 ApoE4-Aβ− patient, and the Aβ status was unknown for 7 patients. These cases were excluded from the relevant calculations and from the figures.

We went on to test whether the modifying effect of ApoE in FTD-tau might relate to the presence of Aβ copathology without meeting criteria for a formal pathological diagnosis of AD in 139 cases with available neuropathological data. Postmortem cases were neuropathologically staged according to Thal phases and Braak and Braak stages [14], [15]. A number of cases (n = 44) scored at Thal phases 0, 1, 2, or 3, but none reached Thal phase 5 or Braak stage 6, needed for a definitive diagnosis of AD. ApoE4 carrier status increased the likelihood of Aβ pathology, both in cases with tau and TDP43 pathology (chi-squared test, *P* = .001 and chi-squared test, *P* = .006, respectively): for the tau+ ApoE4+ patients with FTD and available neuropathology data, 15 of 21 (71.4%) had Aβ copathology compared with 11/42 (26.2%) ApoE4− tau+ cases (chi-squared test, *P* = .001); for the TDP+ ApoE4+ FTD cases, 8/13 (61.5%) had Aβ copathology compared with 10/47 (21.3%) of ApoE4− TDP+ cases (chi-squared test, *P* = .013). The prevalence of Aβ copathology was similar when tau+ cases were compared with TDP+ cases (chi-squared test, *P* = .259) and was associated with ApoE4 carrier status, both in cases with tau pathology (chi-squared test, *P* = .001) and without tau pathology (chi-squared test, *P* = .006). Patients who were positive for Aβ pathology were significantly older at onset than patients who were found to have no Aβ pathology at postmortem (60.9 vs. 57.1 years old, respectively, t-test, *P* = .024). In patients with FTLD-tau, ApoE genotype and Aβ copathology had independent associations with age at clinical onset (linear regression, n = 62, ApoE -10.7 years, *P* = 5.2 × 10^−5^; Aβ +7.6 years, *P* = .002). The effects of ApoE4+ carrier status and Aβ on AAO were independent of each other both in patients with and without tau (univariate ANOVA, *P* = .550 and *P* = .545, respectively).

Our analyses, carried out in a well-characterized cohort of patients with FTD, provide evidence that ApoE4 genotype accelerates neurodegeneration in patients with *MAPT* mutations or FTLD-tau pathology independent of Aβ—notably AAO in FTD-tau cases was on average about 3.9 years earlier in those who carried an ApoE4 allele. Previously, Shi et al. proposed that ApoE4 genotype acts through a toxic gain of function mechanism to exacerbate or modify tau pathology, neuroinflammation, autophagy, and reactive astrocyte activation; they provided extensive evidence in a mouse model of FTD and in neuropathological cases of tauopathies, as well as clinical support in an AD cohort [6]. Our data further strengthens this hypothesis providing evidence in human that ApoE4 modifies the clinical phenotype of FTLD-tau, independent of Aβ copathology. While ApoE4 status was associated with Aβ copathology at postmortem examination in both tau+ and in TDP+ cases of FTD, its effect on AAO was specific to tau+ FTD cases (Fig. 1A). Indeed, ApoE4 and Aβ copathology were associated with opposing effects on AAO in our data set in tau+ FTD cases (Fig. 1B). The confounding impact of Aβ copathology, along with the heterogeneous nature of FTD pathology and the specificity of the effect of ApoE4 on tau pathology may explain why previous studies of ApoE4 in FTD [7], [8], [9], [10] did not detect this influence. Despite the robust statistical finds and the large cohort of patients with FTD, analysis of pathological and gene mutation–defined subgroups inevitably leads to relatively restricted numbers and therefore further replication will be important.

**Research in context**

1. Systematic review: We reviewed the literature searching for “ApoE” and “frontotemporal dementia” or “FTD”, establishing that other than Shi et al*.* (*Nature,* 2017) little information on the effects of ApoE on primary tauopathies and FTD was available. Shi et al*.* established a primary effect of ApoE4 on tau pathology and neurodegeneration, independent of amyloid β (Aβ) pathology.
2. Interpretation: Our data in patients with FTD confirm that ApoE4 exacerbates the disease in patients with or expected to have tau pathology, lowering the age at clinical onset significantly. This effect is more pronounced if confounding effects of Aβ copathology are taken in to account.
3. Future directions: Future studies should analyze the effects of ApoE genotype on FTD, accounting for differential effects on tau and Aβ copathology, to replicate the effects found here. Further *in vitro* studies to elucidate the cellular mechanisms for this effect will be important.
